# Imaging 1927 nm Fractional Thulium Laser‐Tissue Interactions: A Spectrum of Nonablative to Ablative Effects

**DOI:** 10.1111/jocd.70304

**Published:** 2025-07-16

**Authors:** Emily Wenande, Kevin Jacobsen, Gabriela Lladó Grove, Uwe Paasch, Merete Haedersdal

**Affiliations:** ^1^ Department of Dermatology Copenhagen University Hospital – Bispebjerg and Frederiksberg Copenhagen Denmark; ^2^ University of Leipzig Leipzig Germany; ^3^ Department of Clinical Medicine University of Copenhagen Copenhagen Denmark

**Keywords:** histology, laser‐tissue interactions, line‐field confocal optical coherence tomography, microthermal treatment zones, morphology, noninvasive imaging, optical coherence tomography1927 nm laser

## Abstract

**Background:**

Infrared fractional lasers that target tissue water are traditionally divided into nonablative and ablative devices. The 1927 nm fractional thulium fiber laser (FTL) may, due to an intermediate water absorption coefficient, offer a range of nonablative‐to‐ablative effects.

**Objective:**

This study explored dynamic 1927 nm FTL‐tissue interactions produced by different pulse energies in in vivo human skin, using non‐invasive optical coherence tomography (OCT) and line‐field confocal OCT (LC‐OCT) imaging.

**Methods:**

FTL exposure was performed on in vivo antero‐lateral forearm skin at 0, 3, 15, or 20 mJ pulse energy using two separate laser tips (spot size: 200 μm (C1); 350 μm (C5)) at 20‐watt power. Immediately after, LC‐OCT and OCT imaging enabled qualitative description of microthermal treatment zone (MTZ) morphology, as well as semiquantitative measurement of MTZ diameter and ablation depth.

**Results:**

Ranging from nonablative subepidermal effects to frank ablation, imaging revealed a variety of MTZ morphologies depending on pulse energy and laser tip. At low 3 mJ pulse energy, effects were generally limited to the epidermis, with MTZs consisting of subepidermal clefts (C5) or disruptions through the viable epidermis (C1) under a residual stratum corneum. Rising 15–20 mJ pulse energies expanded thermal effects in the lateral and vertical plane, leading to wider MTZs (e.g., C1: 3 mJ vs. 20 mJ: 213 vs. 357 μm), more extensive stratum corneum involvement, and increasing ablation depth to the superficial dermis (e.g., C1: 3 mJ vs. 20 mJ: 93 vs. 101 μm).

**Conclusion:**

Visualized by combined LC‐OCT and OCT imaging, FTL‐tissue interactions are highly modifiable and span the nonablative to ablative spectrum depending on pulse energy and laser tip.

## 1 Introduction

**FIGURE 1:**
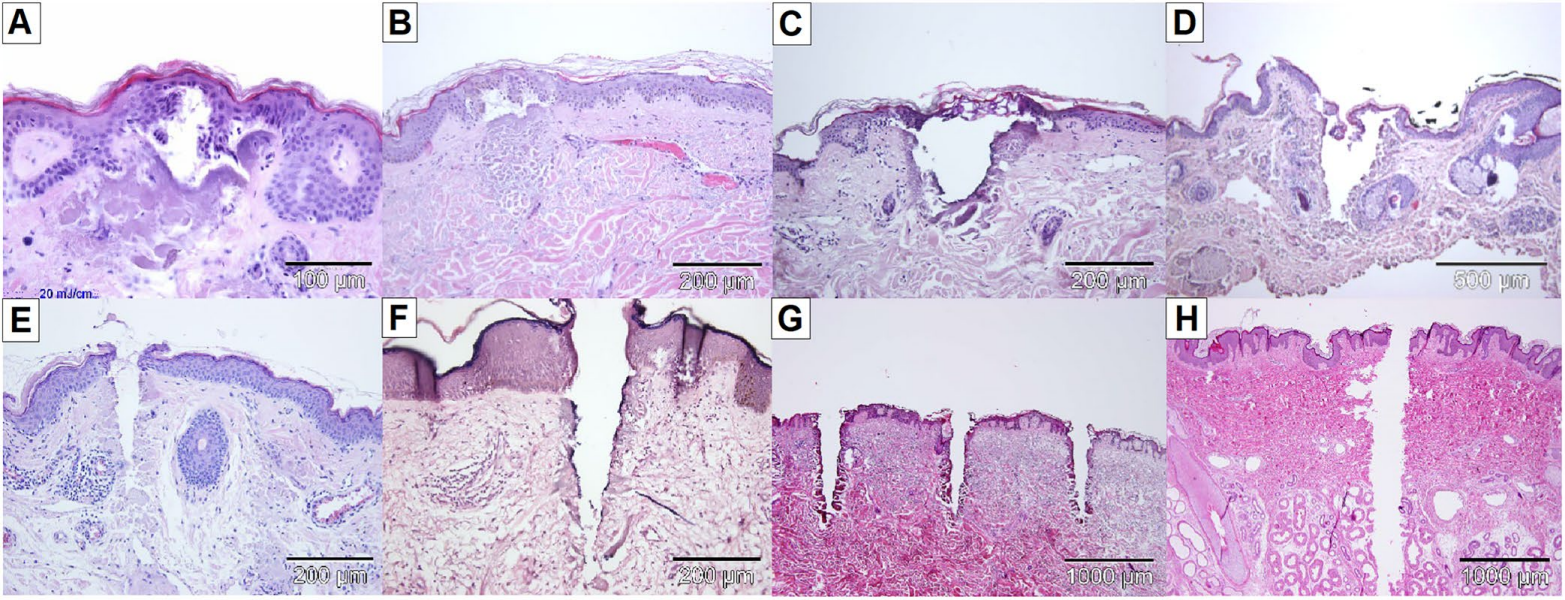
A spectrum of histological laser‐tissue interactions generated by traditional nonablative and ablative fractional infrared lasers. (A) Nonablative effect: Intact stratum corneum, partial disruption of the viable epidermis with subepidermal clefting, superficial dermal coagulation (1550 nm laser, 20 mJ). (B) Nonablative effect: Intact stratum corneum, partial disruption of the viable epidermis with subepidermal clefting, deeper dermal coagulation, coagulated vessels (1550 nm laser, 70 mJ). (C) Semiablative effect: Residual, thermally‐impacted stratum corneum scaffold, ablation of the viable epidermis and superficial dermis, thick coagulation zone (10 600 nm laser, continuous wave (CW), 10 W). (D) Semiablative/ablative effect: Disrupted stratum corneum scaffold, ablation of the viable epidermis and superficial dermis, thick coagulation zone (10 600 nm laser, CW, 20 W). (E) Ablative effect: Complete ablation of epidermal compartment including stratum corneum, ablation within superficial and deep dermis, thick coagulation zone (10 600 nm laser, ultrapulse, 10 mJ). (F) Ablative effect: Complete ablation of epidermal compartment including stratum corneum, ablation within superficial and deep dermis, medium coagulation zone (10 600 nm laser, superpulse, 60 mJ). (G) Ablative effect: Complete ablation of epidermal compartment including stratum corneum, ablation within superficial and middle deep dermis, medium coagulation zone (10 600 nm laser, CW). (H) Ablative effect: Complete ablation of epidermal compartment including stratum corneum, ablation within superficial and very deep dermis, thin to no coagulation zone (10 600 nm laser, ultrapulse).

The development of infrared fractional laser technology in the beginning of the 21st century represents a significant milestone in energy‐based dermatological therapy [1, 2]. By emitting light wavelengths that are preferentially absorbed by water in irradiated skin, these devices produce columns of microscopic thermal injury while sparing surrounding tissue. The nature of photothermal effects is influenced by a specific laser wavelength's water absorption coefficient [3, 4, 5]. Lasers that emit light more weakly absorbed by water (e.g., 1550 nm) are traditionally categorized as “nonablative”, since their thermal effects are primarily coagulative and leave the outermost stratum corneum (SC) intact. In contrast, due to higher water absorption, “ablative” lasers (e.g., 2940 or 10 600 nm) effectively vaporize the superficial skin layers including the SC, leaving varying degrees of residual, peripheral coagulation [3, 4, 5]. The spectrum of microthermal effects produced by these typical nonablative and ablative fractional devices is thus wide‐ranging. As illustrated in Figure 1, histological laser‐tissue interactions can range from discrete dermal or epidermal coagulation with complete preservation of the SC (Figure 1A), to striking full‐thickness ablation (Figure 1H). Effects are further modified by specific laser settings, treatment regimens (i.e., laser handpiece, stacking, and passes) [6], and skin tissue characteristics (i.e., hydration [7], anatomic location [8], healthy versus diseased skin [9] etc.).

Fractional lasers with new, alternative wavelengths are successively being introduced to maximize efficacy and improve side‐effect profiles. Among them, the 1927 nm fractional thulium fiber laser (FTL) is used to treat pigment disorders [10, 11, 12, 13, 14, 15], photodamage and aging skin [10, 16, 17]. The somewhat intermediate 1927 nm wavelength is more greatly absorbed by tissue water than classic nonablative fractional lasers (NAFLs) (e.g., 1540 or 1550 nm), but markedly less than ablative wavelengths such as the 10 600 nm CO_2_ laser [4, 18]. Consequently, FTL's thermal effects are more superficial than NAFLs, primarily targeting the epidermal and superficial dermal compartment, with less impact on the outermost, dehydrated SC than ablative fractional lasers (AFLs) [6]. As such, FTL has the potential to produce microthermal effects at the midpoint between traditional NAFL and AFL spectra. Despite FTL's widespread use in medical and cosmetic settings however, few published studies characterize the device's range of laser‐tissue interactions in in vivo human skin.

Characterization of laser‐tissue interactions is traditionally performed using histological analysis of ex vivo skin samples. Although gold standard, histology is not without limitations, being an invasive nonreal‐time assessment requiring sample processing that can result in tissue shrinkage and distortion [19, 20, 21]. In the last decade, several real‐time noninvasive imaging tools have therefore been introduced as alternatives to histology. Among them, optical coherence tomography (OCT) has emerged as a useful and rapid technique for visualizing laser‐tissue interactions in vivo, capturing morphology of laser‐generated microthermal treatment zones (MTZ) and healing over time [7, 22, 23, 24]. More recently, line‐field confocal OCT (LC‐OCT) was added to the imaging armamentarium for analyzing lasers' immediate and therapeutic effects [25, 26, 27]. The technology offers an optical resolution superior to OCT (~1 μm vs. 7.5 μm), but with inferior depth penetration (0.5 mm vs. 1 mm) [28]. Combined, the capabilities of OCT and LC‐OCT imaging may be complimentary, achieving adequate penetration depth and histology‐like visualization of skin layers ideal for laser‐tissue interaction evaluation. Thus, using the combined capabilities of LC‐OCT and OCT imaging, this study explored the range of 1927 nm FTL‐tissue interactions produced by a variety of pulse energies in in vivo human skin.

## 2 Methods

### 2.1 Study Setup

FTL's range of laser‐tissue interactions was investigated in healthy in vivo human skin using two noninvasive imaging modalities. In practice, eight test sites (15 × 15 mm) on the anterolateral forearm of an informed, consenting volunteer (Fitzpatrick skin type II) were exposed to FTL at four different pulse energies using two different laser tips. Using combined LC‐OCT and OCT imaging, resulting MTZ dimensions, morphologies, and dynamic healing responses were evaluated immediately, as well as up to 30 days after FTL, respectively.

### 2.2 Study Procedures

#### 2.2.1 Fractional Thulium Laser

Laser irradiation was performed using a 1972 nm FTL device (LaseMD, Lutronic, Seoul, South Korea) with two commercially available laser tips (C1 or C5) in static energy modes. The C1 roller‐tip had a 200 μm spot size and a density of 100 spots/cm^2^, whereas the C5 tip had a 350 μm spot size and a density of 225 spots/cm^2^. Each of the eight test areas was exposed to a single pass at 0, 3, 15, or 20 mJ pulse energy at fixed 20‐watt (W) power.

#### 2.2.2 Non‐Invasive Imaging

At the designated time points, LC‐OCT imaging was performed by an experienced user with a CE‐marked, commercially available, hand‐held LC‐OCT instrument (DeepLive, DAMAE Medical, Paris, France). The system uses interferometry, confocal filtering, and a broadband light source centered at 800 nm wavelength to obtain images with an estimated spatial resolution of 1.0 μm. Scans were acquired as three‐dimensional (3D) images (field‐of‐view of 1.2 mm × 0.5 mm × 0.5 mm) enabling visualization of two‐dimensional (2D) images in the cross‐sectional and *en‐face* view [28].

OCT imaging was performed by the same investigator using a commercially available CE‐marked OCT system (VivosightDX, Michelson Diagnostics Ltd., Kent, UK) at a laser wavelength of 1305 nm. The system has an axial resolution of < 10 μm, a lateral resolution of < 7.5 μm and a penetration depth of 1 mm [29]. A multi‐slice modality, consisting of 250 B‐scans, was applied with OCT images (field‐of‐view of 6 mm × 6 mm) presented in both cross‐sectional and *en‐face* views. Test areas were also documented by digital dermoscopy images, obtained using a handheld dermatoscope (FotoFinder Handyscope, Fotofinder Systems GmbH, Bad Birnbach, Germany) attached to a smartphone (Samsung Galaxy S21, 5G, Samsung Electronics GmbH, Schwalbach, Hesse, Germany).

### 2.3 Laser‐Tissue Interaction Evaluations

Range of laser‐tissue interactions was assessed immediately after exposure. For each FTL energy setting, qualitative description of MTZ morphology was performed using both vertical and *en‐face* LC‐OCT and OCT images. To supplement descriptions, mean MTZ diameter and ‐ablation depth (μm) were measured by one unblinded assessor in vertical LC‐OCT images using a semiquantitative approach with proprietary computer‐embedded software (DAMAE Medical, Paris, France). Presented as means and ranges, semiquantitative assessments were based on a total of 18 distinct MTZs examined in 24 LC‐OCT images, representing 3 MTZs per intervention (C1 and C5 each at 0, 3, 15, 20 mJ). Specifically, MTZ diameter was measured at the width of thermal denaturation at its broadest point for each MTZ, while ablation depth represented the distance between the deepest point of FTL‐induced cavitation/cleft and the surface of the SC. Due to loss of signal/shadowing produced by overlying thermally impacted epidermal structures, assessment of MTZ's dermal compartment (i.e., MTZ depth), could not reliably be performed in LC‐OCT or OCT images.

In addition, the spatiotemporal healing process following FTL was qualitatively described, using OCT and LC‐OCT images taken at 1‐, 4‐, 6 h, 24 h, as well as 7‐ and 30 days post‐laser at 0, 3, and 20 mJ.

## 3 Results

### 3.1 Range of 1927 Nm Fractional Thulium Laser (FTL)‐tissue Interactions

**FIGURE 2:**
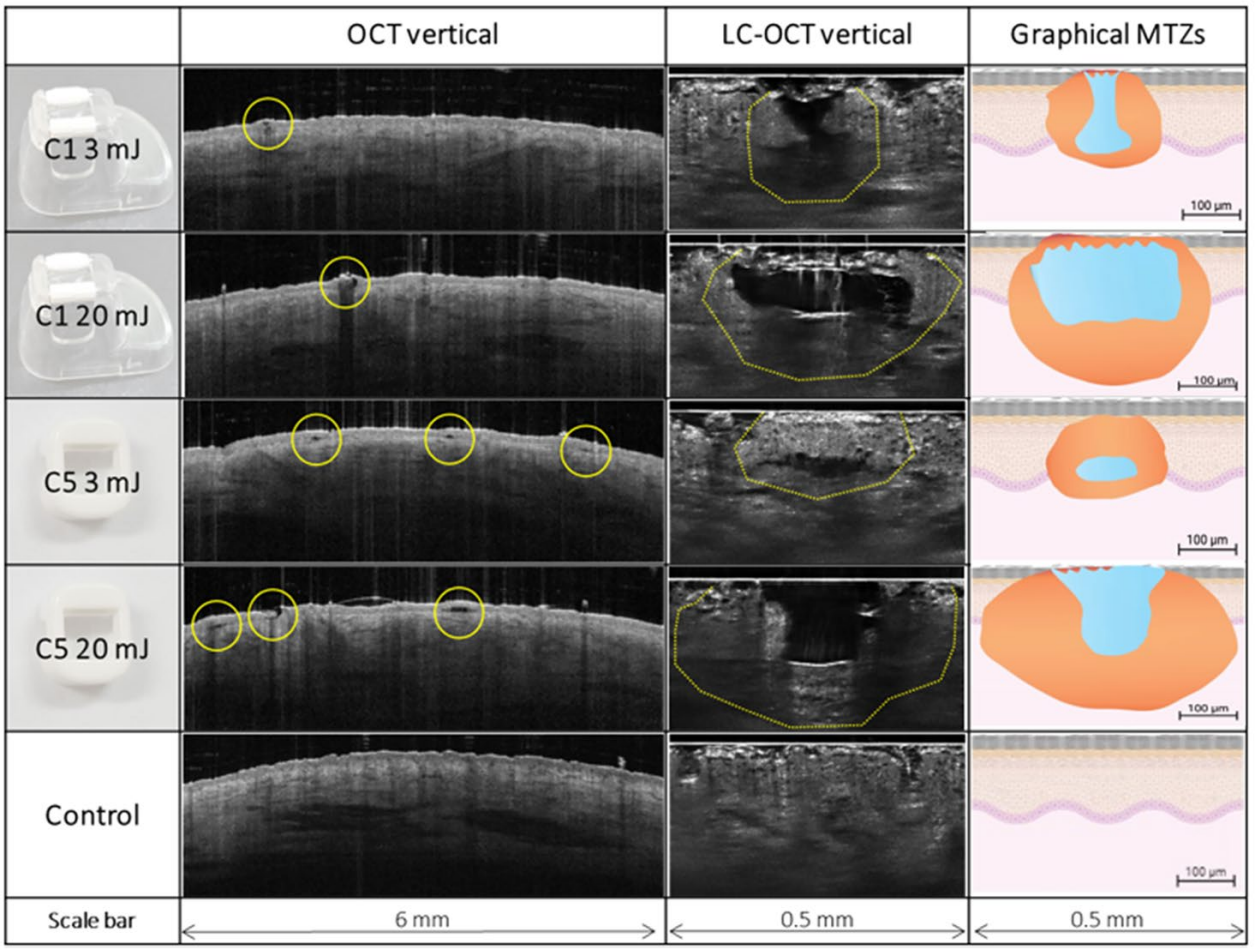
Vertical OCT and LC‐OCT images with supplementary graphical illustrations of microthermal treatment zones (MTZ) immediately after 1927 nm fractional thulium fiber laser exposure at low (3 mJ) and high (20 mJ) pulse energy with two different laser tips (C1 and C5). OCT images show gross morphology of multiple MTZ's (yellow circles); LC‐OCT images depict single MTZ's in high resolution (dashed yellow lines). Graphical illustrations of MTZ's observed in LC‐OCT images are illustrated as single MTZ's representing thermally impacted tissue (orange) with central ablation defects (blue). C1 laser tip: At 3 mJ, incomplete removal of the epidermal compartment is seen with perforation of the viable epidermis and residual, intact stratum corneum; at 20 mJ, increased thermal impact with expanded ablation of the epidermal compartment. C5 laser tip: At 3 mJ, focal subepidermal clefting is seen under a preserved epidermis; at 20 mJ, widened and deeper MTZ with complete poration of the epidermis.

**FIGURE 3:**
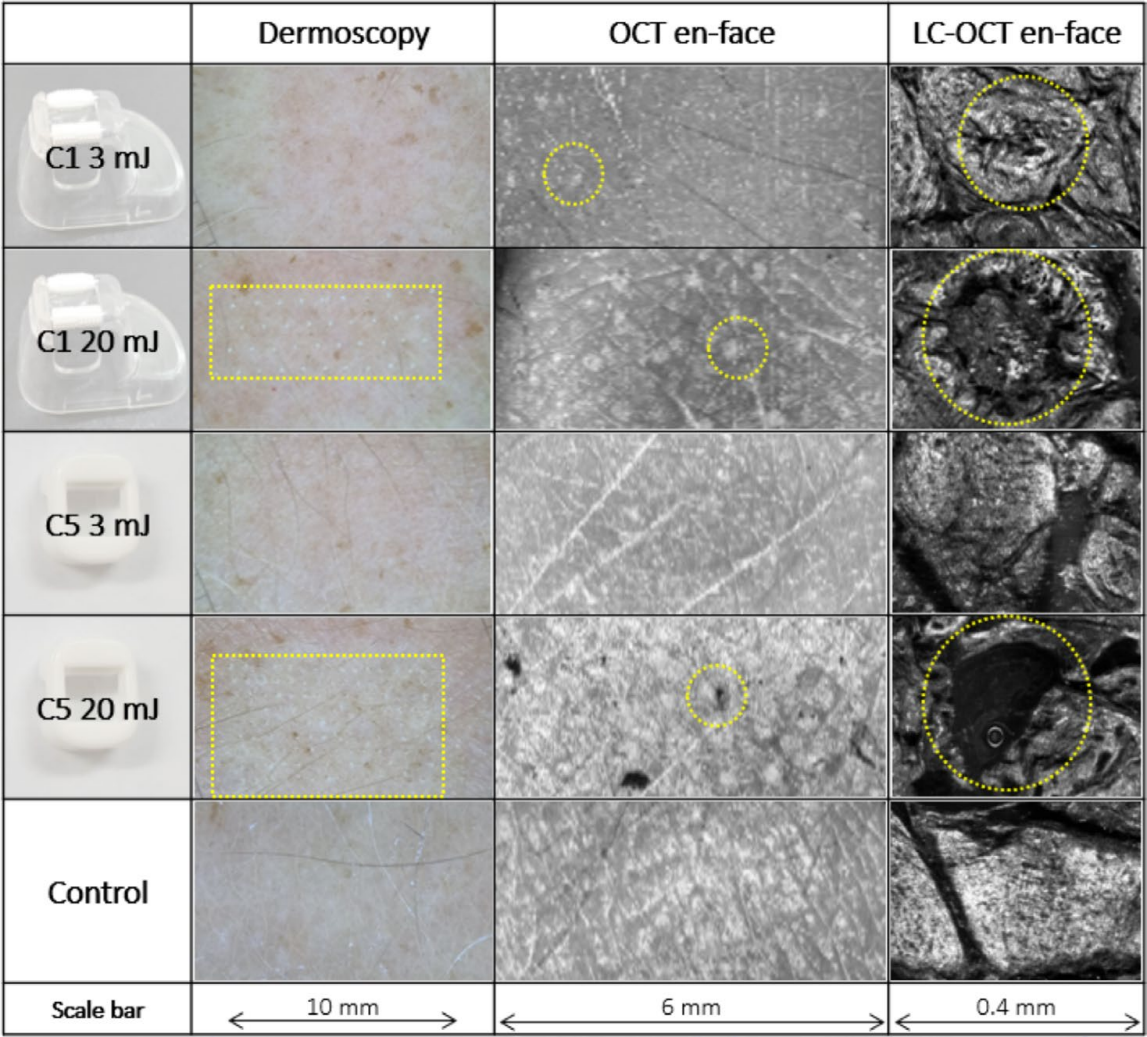
*En‐face* OCT and LC‐OCT images of the skin surface and microthermal treatment zones (MTZ) generated by a 1927 nm fractional thulium fiber laser with low (3 mJ) and high (20 mJ) pulse energy with two different tips (C1 and C5) as well as supplementary dermoscopy images. OCT and LC‐OCT images are captured at the surface of the stratum corneum (SC). C1 laser tip: At 3 mJ, microdisruptions in the SC architecture are seen particularly in LC‐OCT images corresponding to MTZs; at 20 mJ, area of SC disruption expands laterally, forming circular depressions in the surface seen on LC‐OCT. C5 laser tip: At 3 mJ, no thermal impact detected on OCT or LC‐OCT at the SC surface; at 20 mJ, a subset of MTZ demonstrates full poration of the SC surface, detected using both imaging modalities.

Generally, FTL's microthermal effects were superficial, primarily located in the epidermal and superficial dermal compartments. Notable variation in MTZ morphology was observed depending on pulse energy and tip selection, ranging from focal, subepidermal effects to frank ablation (Figures 2 and 3). At low 3 mJ pulse energy, the C1 laser tip created narrow defects beginning at the dermo‐epidermal junction (DEJ) and disrupting the viable epidermis (Figure 2). The overlaying SC scaffold, although grossly intact, demonstrated multiple microdisruptions in its surface shown in LC‐OCT images (Figure 3). Increasing pulse energy expanded thermal damage laterally and profoundly, increasing mean MTZ diameter (3 mJ vs. 20 mJ: 213 vs. 357 μm) and ablation depth toward the superficial dermis (3 mJ vs. 20 mJ: 93 vs. 101 μm) (Table 1).

**TABLE 1:** Semi‐quantitative line‐field confocal optical coherence tomography (LC‐OCT)‐based measurement of microthermal treatment zone (MTZ) diameter and ablation depth generated by fractional thulium fiber laser.

Tip	Pulse Energy (mJ)	MTZ Diameter (μm)*	Ablation Depth (μm)*
C1	3	213 (200–230)	93 (90–95)
	15	353 (350–355)	99 (87–111)
	20	357 (350–370)	101 (100–103)
C5	3	230 (200–250)	75 (70–79)
	15	421 (420–424)	90 (79–101)
	20	433 (420–450)	115 (111–120)

* Data presented as means and ranges, based on measurement of three distinct MTZs per pulse energy (*n* = 3).

A more discrete epidermal impact was seen using the C5 laser tip at low 3 mJ pulse energy. Accordingly, MTZs consisted of focal sub/intraepidermal clefts under an intact epidermal compartment (Figure 2) and an ostensibly preserved SC (Figure 3). Like C1, increasing pulse energy to 15–20 mJ led to wider MTZs (3 mJ vs. 20 mJ: 230 vs. 433 μm) and deeper ablation (3 mJ vs. 20 mJ: 75 vs. 115 μm) (Table 1). In addition, compared to low pulse energy, a greater superficial epidermal impact was also observed, with a minority of MTZs (< 10%) displaying frank poration through the epidermis/SC resembling that of AFL‐produced microscopic ablation zones (MAZ) (Figures 2 and 3).

Directly comparing laser tips, a greater range in MTZ morphology was offered by the C5 tip, as it generated discrete subepidermal clefts as well as full epidermal poration depending on increasing pulse energy (Figures 2 and 3). The C1 tip, on the other hand, produced narrower MTZs than C5, shown consistently at low to high pulse energy (e.g., C1 vs. C5: mean 213 vs. 230 μm) (3 mJ); 357 vs. 433 μm (20 mJ) (Table 1).

### 3.2 Post‐Laser Healing

**FIGURE 4:**
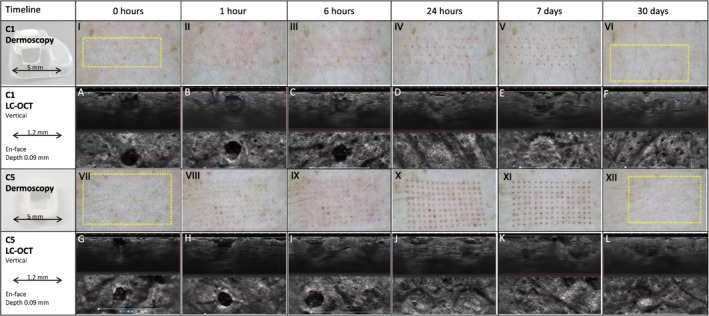
Spatiotemperal healing patterns after 1927 nm fractional thulium fiber laser exposure with high (20 mJ) pulse energy with two different tips (C1 and C5) presented with dermoscopy and LC‐OCT images (vertical and en face). At each time point, different and occasionally multiple MTZs are captured in LC‐OCT images.

Illustrated in Figure 4, a similar healing pattern was observed in all laser‐treated sites. Immediately after FTL, MTZs were seen clearly on LC‐OCT and OCT images. Over the following 6 h, ablation defects began to fill with material, visualized using LC‐OCT as a graying of the hyporeflective cavity (Figure 4C,I). At 24 h, epidermal disruptions had reepithelialized with inwardly migrating keratinocytes creating an epidermal “bridge” (Figure 4J) under newly formed microscopic epidermal necrotic debris (MENDs) (Figure 4D,J) [30]. At day 7, MENDs displayed progressive upward extrusion towards the skin surface on LC‐OCT (Figure 4E,K). Thirty days post‐FTL, the epidermal compartment appeared normalized, with focal dermal remodeling noted under areas where MTZs had formerly been identified (Figure 4F,L).

## 4 Discussion

Understanding energy‐based devices' range of tissue interactions is integral to targeting the right skin compartment, optimizing treatments, and taking full advantage of a given device. In this investigation, LC‐OCT and OCT were used to noninvasively characterize a range of FTL‐tissue interactions in in vivo human skin. Imaging demonstrated notable variation in FTL's microthermal effects depending on pulse energy and laser tip selection. FTL at low pulse energy (3 mJ) produced MTZs that either consisted of subepidermal clefts (C5) or defects that extended through the viable epidermis (C1), coinciding with a spared or only microdisrupted SC, respectively. Increasing pulse energy (15–20 mJ) generally produced wider MTZ diameters and somewhat deeper ablation to the superficial dermis for both tips, with some MTZs demonstrating a fully perforated SC. These observations challenge the common characterization of FTL as an exclusively nonablative device, since not only focal effects at the DEJ but also semiablative and even AFL‐like perforation of the SC are possible depending on parameter selection.

**FIGURE 5:**
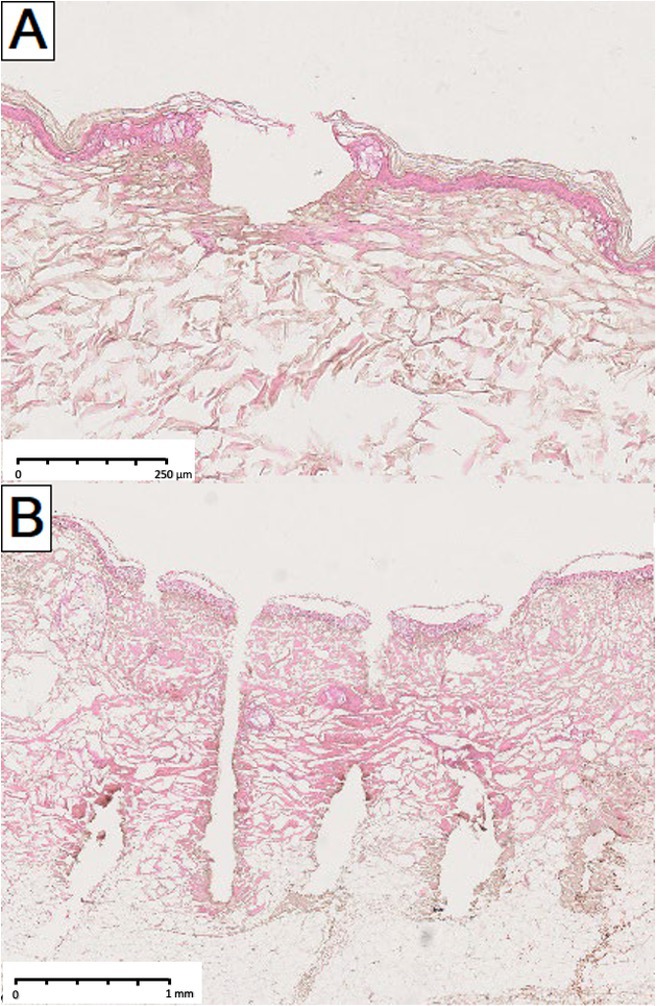
Range of laser‐tissue interactions produced by a 10 600 nm ablative fractional CO_2_‐laser at gentle and aggressive settings. (A) Disrupted residual stratum corneum scaffold, ablation of the superficial dermis with a thick surrounding coagulation zone (chopped continuous wave, 0.012 J/spot, 0.6 ms, 20 W). (B) Complete ablation of epidermal compartment, superficial‐ and very deep dermis with a thin surrounding coagulation zone (superpulse mode, 0.18 J/spot, 3 ms, 60 W).

Our findings highlight the broader point that setting selection, including pulse energy, can powerfully modify the microthermal effects of a given laser. Certainly, a spectrum of MTZ morphologies can also be generated by other fractional infrared lasers at gentle or aggressive settings (Figures 1 and 5). For the FTL studied here, our findings suggest a microthermal spectrum that spans the nonablative to ablative range, including semiablation (i.e., disruption of the residual SC scaffold and subcorneal tissue vaporization) and even full MAZ‐like poration (though only a subset < 10% of C5 MTZs at maximum pulse energy). The FTL is not unique in this regard; a similar phenomenon of different nonablative and ablative effects is also described for other water‐targeting lasers, as well as for 1064 and 532 nm picosecond lasers depending on pulse energy [31, 32]. Such evidence highlights that a one dimensional characterization of a device as “nonablative” or “ablative” runs the risk of over‐simplification. Generally, laser systems should not be understood as “one‐trick ponies”.

Mirroring our imaging results, previous histological analyses describe FTL's wide and superficial MTZs with well‐defined intraepidermal or subepidermal clefting over an SC scaffold [33, 34, 35]. Although some studies suggest that the upper epidermis remains intact, our findings indicate that distruction of the viable epidermis is possible even at low pulse energy (Figure 2, 3 mJ, C1 tip). These shallow but semi‐ablative epidermal effects explain why 1927 nm lasers are well‐suited for treating epidermal and DEJ processes, including actinic keratoses [3, 17] and various pigment disorders [10, 11, 12, 13, 14, 15]. Our images furthermore display alternative forms of ablation that, while leading to loss of underlying skin tissue, do not result in SC removal (Figures 1C and 2).

Intriguingly, thermal impact of the SC was detected with most of the applied settings in this FTL study. Indeed, *en‐face* LC‐OCT imaging revealed microdisruptions of the SC surface even at low pulse energy (Figure 3, 3 mJ, C1 tip) that were not detected by OCT. This finding is supported by two studies of 1927 nm laser‐tissue interactions that used scanning electron microscopy: at low pulse energy (3–5 mJ; 5 W), these studies also observed “multiple, small round holes in the SC surface”, interpreted as tortuous micropores generated by water vaporization [33, 36]. At high 20 mJ pulse energy, MTZs presented on the SC surface as deflated “dome‐shaped papular zones” [33]. These findings bear striking resemblance to the circular SC depressions seen in *en‐face* LC‐OCT images in our study (Figure 3, C1). The finding that FTL impacts the structure of the SC is clinically important. In dermatological settings, 1927 nm devices have been used for topical laser‐assisted drug delivery (LADD) in the context of photodynamic therapy [35], treatment of melasma [37, 38], photoaging and hair loss [33]. These drug delivery studies illustrate FTL's *functional disruption* of the skin barrier. Correspondingly, our images clearly visualize examples of SC microchannels, as well as ablation of the viable epidermis and superficial dermis, underpinning why the 1927 nm FTL has a broader range of modifiable laser‐tissue interactions for LADD compared to classical NAFLs, which traditionally are primarily coagulative and leave the SC intact [18, 39, 40]. Given that thulium parameter optimization for LADD largely depends on SC disruption, we expect augmented drug delivery even at lower FTL settings, based on microdisruptions in the SC architecture observed in our study at 3 mJ pulse energy. However, these effects may vary depending on anatomical site, skin hydration, and disease states, which affect SC thickness [41].

In the literature, FTL's thermal penetration depth (i.e., MTZ depth) is generally quoted as 200–300 μm, reaching the papillary or superficial dermis [12, 16, 42]. Since the image quality needed to precisely measure dermal denaturation (i.e., MTZ depth) was not provided by LC‐OCT or OCT in this study, we instead determined FTL's *ablation/clefting* depth. Using LC‐OCT, we found maximum mean depths of 101 and 115 μm for C1 and C5 tips, respectively. The corresponding MTZ depth is expected to be 100–200 μm deeper, based on a previous histological report that specifically measured dermal penetration of FTL [34]. Our imaging‐based results thus align well with available histological evidence on FTL. The relationship between ablation depth and pulse energy showed a different pattern than is typical for other fractional infrared lasers [21, 43]. A study of the 1540 nm laser, for example, found that a 10 mJ increase in pulse energy enhanced penetration depth with a factor of 100–150 μm [43]. This linear depth‐to‐energy profile was not observed in our study and is consistent with other reports on FTL systems [3, 6, 39, 44].

Dynamic wound healing following FTL exposure is described in two previous histological studies [33, 36]. Generally, MEND formation is seen one day post‐laser, with complete epidermal reepithelization occurring at 1–3 days [36]. By day 7, MENDs appear toward the SC surface and are later exfoliated, while clefts remain visible at the DEJ [33, 36]. Dermal modeling is ongoing throughout the process. In our study, each of these spatiotemporal phenomena were accurately captured by combined LC‐OCT and OCT imaging (Figure 4). The imaging modalities thus appear to be useful alternatives to histology for real‐time assessment of superficial healing responses post‐laser.

Overall, the study revealed various strengths and weaknesses of LC‐OCT and OCT technologies. Among LC‐OCT's strengths was the modality's high resolution, which enabled semi‐quantitative measurement of MTZ dimensions, clear visualization of MTZ morphology including SC architecture, as well as spatiotemporal healing. Weaknesses of LC‐OCT were, however, it's sensitivity to imaging shadowing produced by overlying thermally‐impacted epidermal structures (i.e., which precluded accurate measurement of MTZ depth using the propieraty software), as well as its inability to visualize more than 1–2 MTZs at a time. OCT, on the other hand, offered an overview of the laser grid quickly and with visualization of multiple MTZs simultaneously. Since OCT's resolution was lower, however, assessment of SC integrity was limited and measurement of MTZ dimensions deviated from LC‐OCT‐derived results (data not shown).

The study has some limitations. FTL‐tissue interactions were assessed in a single individual, in one anatomical location (i.e., forearm), and LC‐OCT measurements were based on only three MTZs. While no studies to date have utilized LC‐OCT for real‐time visualization of laser‐tissue interactions in in vivo human skin following thulium laser application, it is important to consider that a larger sample size and inclusion of additional anatomical regions (such as the face) could impact the generalizability of our findings. Moreover, a side‐by‐side comparison between LC‐OCT imaging and histology was outside the scope of the present study. For reference, we have previously demonstrated corresponding intraepidermal clefting and stratum corneum disruption in OCT and histological sections from the same area in porcine skin using similar 1927 nm parameters [35]. Rather than examining the *full* range of FTL effects varying not only pulse energy but power, we elected to examine a single pass at fixed 20 W. This maximal power setting may not be typical for treatment of some indications. Furthermore, different MTZs were assessed in LC‐OCT images at each time point, introducing some uncertainty due to biological variation in microthermal effects. Study strengths, on the other hand, included the multiple pulse energies and timepoints examined, providing a picture of the range of dynamic FTL‐tissue interactions at high power in the in vivo setting. The multimodality approach of combining LC‐OCT, OCT, and dermoscopy allowed for observations detected by one technique to be verified by another, such as MEND location or SC poration.

## 5 Conclusion

Visualized by OCT and LC‐OCT imaging, FTL's microthermal effects are highly modifiable with the potential for focal subepidermal clefting as well as more extensive disruption of the viable epidermis and stratum corneum. As such, rather than categorizing FTL as an exclusively nonablative laser, FTL may be more accurately described as a semi‐ablative device capable of NAFL‐ and AFL‐like effects, depending on setting‐ and laser tip selection.

## Author Contributions

E.W., K.J., G.L.G., and M.H. contributed to the study conception and design. Material preparation, data collection and analysis were performed by E.W., K.J., G.L.G., U.P., and M.H. The first draft of the manuscript was written by E.W. and all authors commented on previous versions of the manuscript. All authors read and approved the final manuscript.

## Consent

Oral and written consent was obtained for patients included in the study.

## Conflicts of Interest

With no relation to the submitted work, Dr. Wenande has received a speaker honorarium from Sanofi, and became a Novo Nordisk A/S employee in September 2024. With no relation to the submitted work, Dr. Paasch has received equipment from Fotona, GME Medical, Alma Lasers, Zimmer, Canfield, FotoFinder, research grants from Zimmer, and consulting fees/speaker honorarium from Alma Lasers, Fotona, Landsberg, Galderma, DDL, DGPRAEC, and Quintessenz publishing. Dr. Haedersdal received the study's laser equipment from Lutronic. With no relation to the submitted work, Dr. Haedersdal has received speaker honoraria and research grants from Galderma, speaker and consulting honoraria from L'Oreal/La Roche‐Posay, research grants from LEO Pharma and a speaker honorarium from Sanofi. Drs. Jacobsen and Grove have nothing to declare.

## Data Availability

The data that support the findings of this study are available on request from the corresponding author. The data are not publicly available due to privacy or ethical restrictions.
